# Deep learning-based detection and classification of airway nodules on CT for early medical intervention

**DOI:** 10.1186/s13244-026-02446-7

**Published:** 2026-11-05

**Authors:** Shulin Liu, Ping Yi, Yaheng Fan, Jing Hu, Chao Zheng, Peng Lei, Sai Li, Fangxue Yang, Chenyu Li, Yangzhen Hou, Yuxin Li, Guanghua Luo, Xiangjun Fang, Haijun Zheng, Harrison Bai, Hui Zhou, Weihua Liao

**Affiliations:** 1 Department of Radiology, The Affiliated Nanhua Hospital, Hengyang Medical School, University of South China, 421002 Hengyang, Hunan China; 2 Department of Radiology, Hunan Engineering Research Center for Intelligent Medical Imaging, Xiangya Hospital of Central South University, No. 87 Xiangya Road, 410008 Changsha, Hunan China; 3 National Clinical Research Center for Geriatric Diseases, FuRong Laboratory, Xiangya Hospital of Central South University, 410008 Changsha, Hunan China; 4 Shukun Technology Co., Ltd, 100102 Beijing, China; 5 Department of Radiology, The First Hospital of Changsha, 410005 Changsha, Hunan China; 6 Department of Radiology, The First Affiliated Hospital of University of South China, 421001 Hengyang, Hunan China; 7 Department of Radiology, The Second Affiliated Hospital of University of South China, 421001 Hengyang, Hunan China; 8 Department of Radiology, Chenzhou First People’s Hospital, 423000 Chenzhou, Hunan China; 9 Department of Radiology-Diagnostics, School of Medicine, University of Colorado Anschutz Medical Campus, Aurora, CO USA

**Keywords:** Airway nodule, Early detection, Classification, Deep learning, Computed tomography

## Abstract

**Objectives:**

Airway lesions are common CT findings in interval and post-screen lung cancers; however, airway nodules are frequently overlooked. Robust artificial intelligence (AI) tools for characterizing airway nodules remain limited. This study aimed to develop and evaluate deep learning-based models for the detection and classification of airway nodules on low-dose CT(LDCT) as a second-reading tool.

**Materials and methods:**

1813 segmental and more proximal airway nodules were retrospectively identified from 1107 LDCT of 1043 participants. A sphere-based center-points matching network, first used to establish a lung nodule detection model, was transferred to airway nodules and enhanced by incorporating coordinate and multiscale attention. The model was trained on 907 proximal airway nodules and independently tested on 738 proximal nodules across four hospitals. The clinical utility of the AI tool was evaluated through human-AI interaction. For nodule classification, a 3D SEResNeXt101-based model was trained, validated and tested using 286 airway nodules (121 benign, 165 malignant) with a 6:2:2 split and evaluated in an independent testing dataset.

**Results:**

On the independent testing set, the detection model achieved a high sensitivity of 73.7%. Human-AI interaction demonstrated AI assistance significantly improved radiologists’ performance, increasing sensitivity from 39.5% to 72.9% and F1-score from 49.4% to 77.2%, while reducing diagnosis time from 83.8 s to 61.8 s. The classification model showed high sensitivities of 82.1%, 71.4%, 94.3%, 72.7% and specificities of 62.7%, 73.9%, 73.9%, 87.5% across multicenter training, validation, testing and independent testing sets, respectively.

**Conclusion:**

This AI system significantly enhances the detection and classification of proximal airway nodules, guiding early medical intervention.

**Key Points:**

***Question*** Improved early detection and classification of airway nodules are critical for lung cancer mortality reduction, but AI-based CAD systems targeting airway nodules remain scarce.

***Findings*** The DL models for detection and classification of proximal airway nodules on chest CT were developed and assisted radiologists to achieve higher detection accuracy.

***Critical Relevance Statement*** The clinical applications of the proposed DL-based system for detecting and classifying proximal airway nodules on chest CT scans help radiologists reduce missed/delayed diagnoses and shorten diagnostic time as a second-reading tool, guiding medical intervention and improving clinical outcomes.

**Graphical Abstract:**

**Figure:**
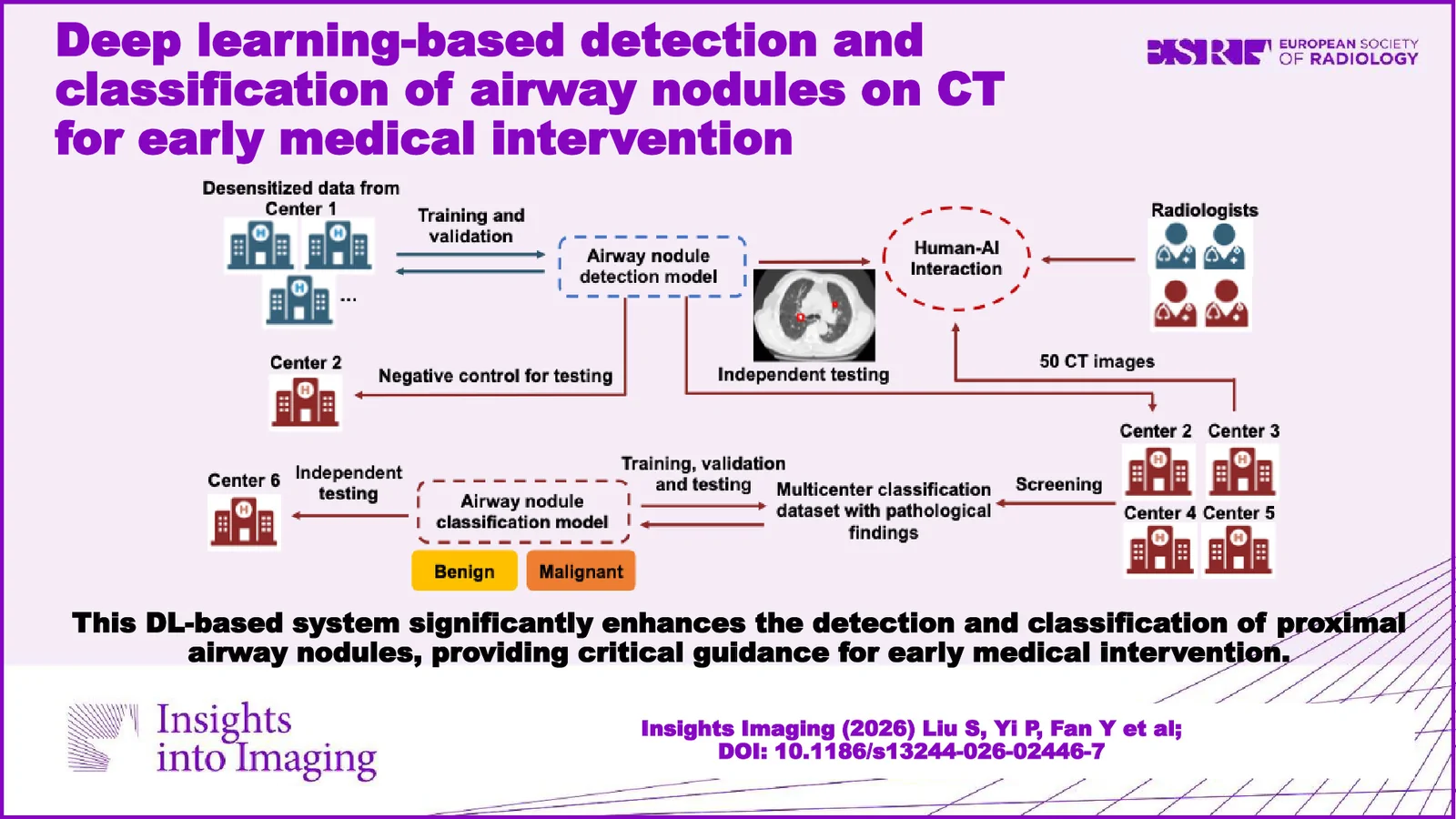

## Introduction

Lung cancer is the most prevalent and life-threatening malignancy worldwide [1, 2]. Early detection of suspicious lung and airway nodules plays a critical role in reducing lung cancer-related mortality [3]. Global cancer mortality statistics from 2022 revealed that China’s age-standardized mortality rates for lung cancer were higher than the global average-127.5 versus 109.9 per 100,000 person-years in males, and 67.8 versus 63.5 in females [1]. Notably, the proportion of patients diagnosed at stage I in China remains lower than that in the United States (17.3% vs. 25.3%) [4], highlighting the urgent need for improved early detection of lung and airway nodules.

Low-dose computed tomography (LDCT) has emerged as a noninvasive and reliable modality for increasing the detection of stage I lung cancer and ultimately reducing cancer mortality [5, 6]. The widespread application of LDCT has led to a substantial increase in the detection of lung nodules [7]; however, accurate identification and characterization of screen-detected airway nodules remain limited and challenging. Given the small size of airway nodules, manual identification by radiologists is often labor-intensive and time-consuming, and is prone to missed diagnoses.

Airway nodules are associated with a broad spectrum of pathological conditions, including benign and malignant tumors, transient secretions, infection, and foreign bodies [8–11]. Among these, tracheobronchial tumors are particularly rare, accounting for approximately 0.4% of all body cancers [10]. Kim et al reported that among 53,036 Korean individuals undergoing LDCT for lung cancer screening (LCS), 313 (0.6%) presented with airway nodules, with follow-up CT/bronchoscopy revealing no malignant lesions in 186 cases (59.4%) [11]. Mendoza et al identified 46 airway nodules (3.5%) among 1297 Lung-RADS category 3 or 4 nodules in 1139 LCS patients, of which 45 (97.8%) were confirmed as benign lesions [12]. These findings indicate that malignant airway nodules detected during LCS are relatively uncommon; however, they carry a substantial risk of progressing to primary squamous cell carcinoma (SCC), and represent one of the most frequently missed findings on CT [10, 13]. White et al found that 10 of 15 (67%) initially missed lung cancer cases on CT scans presented as endobronchial lesions [14]. Similarly, analyses from the NELSON trial demonstrated that 8.9% of interval and post-screen cancers were missed due to errors of detection and interpretation, with 22.7% initially manifesting as central endobronchial nodules [15, 16], highlighting a critical gap in current screening practices.

The Lung Imaging Reporting and Data System (Lung-RADS) version 2022 introduced refined classification criteria for airway nodules based on morphology, number, location and persistence, aiming to standardize the detection and diagnosis [17]. However, manual CT detection remains inadequate for the early detection and risk stratification of airway nodules, due to the neglect of the lung bronchial tree and interpreter fatigue from radiologists [14, 16]. These limitations often lead to delayed interventions, underscoring the urgent need for developing reliable and robust computer-aided diagnosis (CAD) systems to facilitate accurate detection and risk classification of airway nodules, while reducing diagnostic delays, misdiagnoses, and unnecessary follow-up procedures, including invasive bronchoscopy.

Driven by advances in computer technology and the availability of large-scale medical datasets, artificial intelligence (AI) has been widely adopted in medicine and healthcare, particularly for pulmonary nodule detection and risk assessment [18–20]. However, AI-based CAD systems specifically targeting airway nodules remain scarce. A single-center retrospective study initially proposed a CT-based AI system for airway nodule detection in LCS, achieving a sensitivity of 75.1%; however, its clinical applicability was limited by a small training cohort, the absence of external validation, and the lack of a risk stratification model [21]. Most critically, the impact of integrating CAD into routine clinical practice on radiologists’ performance has not been systematically evaluated.

To directly address this unmet need, we developed deep learning (DL)-based models for automated detection and risk stratification of segmental and more proximal airway nodules using multicenter chest LDCT datasets, and evaluated radiologists’ diagnostic performance with and without AI assistance through a human-AI interaction framework. Our results demonstrate that the proposed models significantly enhance radiologists’ interpretive performance for proximal airway nodules, highlighting their potential for clinical implementation to facilitate timely diagnosis and medical intervention.

## Materials and methods

### Datasets

#### Participants

The inclusion criteria refer to patients with segmental and more proximal airway nodules/lesions detected by thin-slice chest CT scans (layer thickness ≤ 5 mm). Given that pulmonary SCC often exhibits a creeping growth pattern, airway lesions with non-nodular manifestations such as wall thickening were also included. The exclusion criteria included: (1) receiving pneumonectomy before inclusion, (2) the endobronchial lesions involving lung parenchyma, and (3) poor quality of chest CT imaging for diagnosis.

Six datasets were retrospectively collected. Dataset 1 comprised chest CT scans of 926 patients from desensitized data collected by Shukun Technology company (Center 1) during 2020-2023. Dataset 2 was obtained from Xiangya Hospital of Central South University (XYHCSU, Center 2) and the Second Affiliated Hospital of University of South China (SAHUSC, Center 3) during 2013-2023. A total of 1487 patients undergoing chest CT scans were identified through manual retrieval in the report impression of Picture Archiving and Communications Systems (PACS) at Centers 2 and 3 using keywords “bronchial lumen” or “bronchial wall” or “endobronchial”. Dataset 3 included chest CT images from 28,033 patients with pathologically confirmed benign/malignant lung neoplasms/lesions from four hospitals in Hunan Province, including Centers 2 and 3, Chenzhou First People’s Hospital (CFPH, Center 4) and the First Hospital of Changsha (FHC, Center 5) during 2013–2023, without overlap with Dataset 2. Malignant cases mainly included lung SCC and sarcoma, while benign cases primarily comprised pulmonary hamartoma, leiomyoma and inflammatory myofibroblastic tumor and pulmonary granuloma. Dataset 4 contained 106 chest CT scans from Center 2 from Jan 2022 to Oct 2022 showing mucous plugging that decreased or resolved on follow-up CT imaging, and was verified according to Lung-RADS v2022 criteria (the presence of air or no soft-tissue density). Dataset 5 consists of 136 chest CT images from 136 participants of Center 2 without airway nodules. Dataset 6 included chest CT images of 62 patients with pathologically confirmed benign/malignant lung neoplasms/lesions or mucous plugging from the First Affiliated Hospital of University of South China (FAHUSC, Center 6) during 2018-2025. Construction and labeling of the six datasets were shown in Table S1.

According to the inclusion and exclusion criteria, 436 chest CT examinations containing 907 segmental and more proximal airway nodules from dataset 1 were screened for training and cross-validation of the nodule detection model. 367 chest CT scans of 303 participants from the datasets 2 and 3 were used as the independent testing dataset for the nodule detection model, containing 263 CT data of 212 patients from Center 2, 71 CT data of 67 patients from Center 3, 8 CT data of 7 patients from Center 4, and 25 CT data of 17 patients from Center 5. Moreover, dataset 5 was applied as a negative control set for testing the nodule detection model.

Regarding nodule classification, 180 CT data containing 180 segmental and more proximal airway nodules from 123 pathologically confirmed patients with benign/malignant lung neoplasms were screened from dataset 3. In addition, 106 cases of mucous plugging from dataset 4 were added to balance the ratio of benign and malignant lesions. Finally, 286 chest CT scans of 229 patients with 121 benign and 165 malignant airway nodules were enrolled from the datasets 3 and 4, and divided into the training, validation and testing sets of the nodule classification model according to the ratio of 6:2:2, respectively. Additionally, 62 chest CT scans of 62 patients with 40 benign and 22 malignant segmental and more proximal airway nodules from dataset 6 were used as the external independent testing dataset. Note that the same patients with multiple CT scans at different ages were counted separately, while repeated scans at the same age were counted once. Multiple nodules from the same patient would not appear simultaneously in the training dataset and testing dataset.

**Fig. 1:**
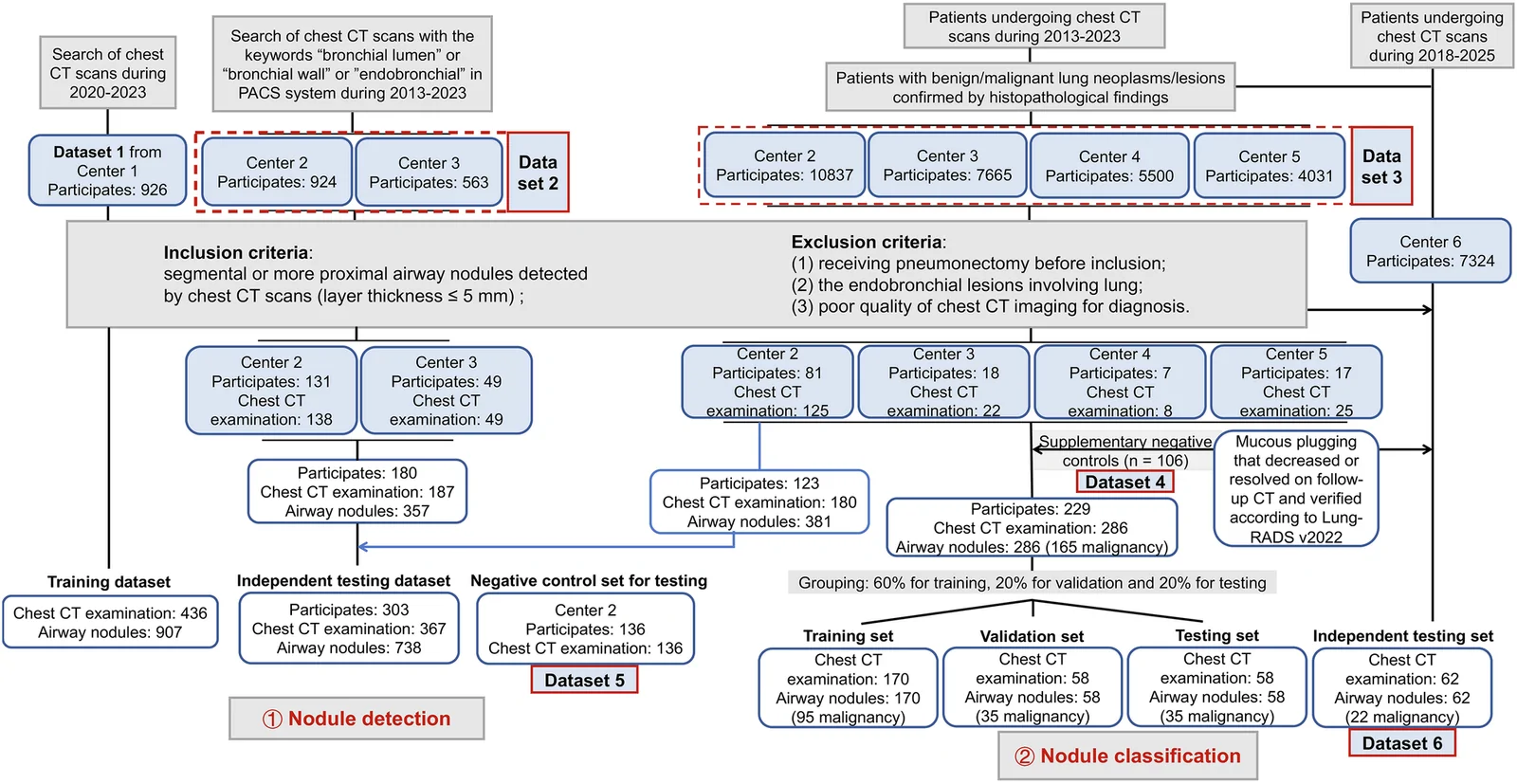
Flowchart for selection of segmental or more proximal airway nodules for nodule detection and classification models

Ethical review was approved by the Ethics Committee of Center 2 (No. 202308742), Center 3 (No. 2023K0227027), Center 4 (No. 2025-018), Center 5 (No. 2023-106) and Center 6 (No. 2022ll0113001), and informed consents were waived. The flowchart of nodule selection for detection and classification models is shown in Fig. 1.

#### Chest CT examination

All patients underwent chest CT scans before pneumonectomy. CT equipment and parameters of scanning and reconstruction across the five hospitals were detailed in Table S2. All the CT examinations were chest LDCT scans without contrast enhancement. Digital imaging and communications in medicine (DICOM)-format chest CT scan images were initially annotated by two junior radiologists with 3 years of experience using Shukun LungDoc (LungDoc; ShuKun Network Technology, Beijing, China; https://en.shukun.net), then verified by an intermediate radiologist with over 5 years of experience and a senior radiologist with over 10 years of experience, generating a gold standard box of airway nodules.

### Establishment of the detection model

The modeling approach of the proposed nodule detection tool consisted of several distinct stages. First, airway segmentation was performed using WingsNet [22]. Subsequently, a nodule detection model was established based on the sphere-based center-points matching network (SCPM-Net) [23], incorporating coordinate attention [24] and multiscale attention [25]. The model was initially pretrained on lung parenchymal nodules, and then adapted for airway nodule localization via deep transfer learning using Joint Adaptation Networks (JAN) [26]. Finally, the model estimates the size and location of detected nodules by outputting a bounding box. Note that a 5-fold cross-validation at the patient level on the training dataset was applied. In each fold, 80% of the patients were assigned to the training set, while the remaining 20% served as the internal validation set to monitor performance, tune hyperparameters, and execute early stopping. A Non-Maximum Suppression (NMS) ensemble strategy was employed for the evaluation on the independent testing dataset. Implementation details appeared in Supplemental materials, and the network architecture of SCPM-Net was illustrated in Fig. S1a.

### Establishment of classification model

The nodule classification model was constructed using the three-dimensional (3D) Squeeze-and-Excitation (SE) ResNeXt101 architecture (Fig. S1b) [27, 28]. The input patches (224 × 224 × 224 voxels) were extracted from the CT scans, centered on the annotated airway nodules. To improve robustness and generalization, data augmentation was implemented, including random 90-degree rotations, sagittal and coronal spatial flips, and the addition of zero-mean Gaussian noise to 30% of training samples.

### Human-AI interaction

50 CT images from 50 patients were randomly selected from the independent testing dataset of the nodule detection model. Readers were recruited from Centers 2 and 3, including two junior radiologists with 3 years of experience and two senior ones with over 10 years of experience. They were not involved in data acquisition and image annotation, and independently reviewed these chest CT scans with or without the assistance of a nodule detection model, with an interval of 3 weeks for cleaning. During the AI-assisted reading session, the radiologists were provided with bounding boxes generated by our detection model superimposed on the CT images. They were informed that the AI model output the location and size of potential airway nodules as a reference before the reading with the assistance of the detection model. The time consumption per patient in the two readings was recorded for efficiency analysis. The order in which CT images appeared in the two readings was different.

### Statistics

Statistical analysis was conducted using R software (version 3.5.0; R Foundation for Statistical Computing), SPSS Statistics (version 26.0; IBM), and Python (version 3.10; Python Software Foundation). Differences in characteristics across datasets were assessed using the Kruskal-Wallis test for continuous variables and the *χ*^2^ test for categorical variables.

All quantitative data were presented as mean ± standard deviation (SD). Sensitivity (SEN), F1-score, false-negative rate (FNR) and 95% confidence intervals (95% CIs) were calculated for the nodule detection model based on lesion-level detection. Given the volumetric nature of 3D bounding boxes, the Intersection over Union (IoU) threshold of 0.1 could ensure a physical intersection between the prediction and the actual lesion, and was used to define a true positive prediction. An area under the Receiver Operating Characteristic curve (AUC), sensitivity (SEN), specificity (SPC), false positive rates (FPR), false negative rates (FNR), positive prediction value (PPV), negative prediction value (NPV), accuracy (ACC), and 95% CI were calculated for the nodule classification model. AUCs were calculated and compared using DeLong’s test, and the optimal cut-off value was determined with the maximum Youden index. Two-sided *p* < 0.5 was considered indicative of a statistically significant difference.

## Results

### Participants and airway nodule characteristics

The training dataset for the nodule detection model comprised 436 participants (mean age, 63.58 ± 11.57 years; 327 male) from Center 1. For the independent testing, 303 patients (mean age, 60.43 ± 11.39 years; 266 male) with 367 chest CT scans were recruited from four hospitals in Hunan Province (Centers 2, 3, 4 and 5) during 2013-2023. In terms of the negative control set, 136 participants (mean age, 34.2 ± 8.4 years; 84 male) with 136 chest CT scans without airway nodules were collected from Center 2. For classification model, 229 participants (mean age, 59.76 ± 11.81 years; 179 male) with 286 chest CT scans during 2013-2023 were enrolled from the above four hospitals as training, validation and testing sets, and 62 participants (mean age, 63.61 ± 11.71 years; 42 male) with 62 chest CT scans from Center 6 during 2018-2025 were enrolled as the external independent testing set (Table 1 and Fig. 1).

**Table 1:** Demographics of participants and characteristics of airway nodules in the training and independent testing datasets for nodule detection, as well as the multicenter classification dataset and independent testing set for nodule classification

Parameters	Training dataset	Independent testing dataset	*p-*value	Multicenter classification dataset						Independent testing set	^*a*^*p* value
				Total	Benign	Malignant	Training set	Validation set	Testing set		
Number of participants	436	303	N/A	229	120	109	150	54	54	62	N/A
Number of CT scans	436	367	N/A	286	121	165	170	58	58	62	N/A
Age (Mean ± SD, years)	63.58 ± 11.57	60.43 ± 11.39	< 0.001	59.76 ± 11.81	54.89 ± 14.46	63.39 ± 7.80	60.87 ± 10.18	57.93 ± 13.57	58.31 ± 14.02	63.61 ± 11.71	0.007
Gender (*N*, %)			< 0.001								0.088
Male	327 (75)	266 (87.8)		179 (78.2)	78 (65)	101 (92.7)	117 (78)	43 (79.6)	46 (85.2)	42 (67.7)	
Female	109 (25)	37 (12.2)		50 (21.8)	42 (35)	8 (7.3)	33 (22)	11 (20.4)	8 (14.8)	20 (32.3)	
Number of nodules	907	738		286	121	165	170	58	58	62	
Location (*N*, %)			< 0.001								0.030
Trachea	249 (27.5)	134 (18.2)		0 (0.0)	0 (0.0)	0 (0.0)	0 (0.0)	0 (0.0)	0 (0.0)	1 (1.6)	
Main bronchus	193 (21.3)	120 (16.3)		11 (3.8)	2 (1.7)	9 (5.5)	6 (3.5)	3 (5.2)	2 (3.4)	6 (9.7)	
Lobar bronchus	186 (20.5)	188 (25.4)		77 (27.0)	8 (6.6)	69 (41.8)	43 (25.3)	12 (20.7)	22 (37.9)	18 (29.0)	
Segmental bronchus	279 (30.7)	296 (40.1)		198 (69.2)	111 (91.7)	87 (52.7)	121 (71.2)	43 (74.1)	34 (58.7)	37 (59.7)	
Size (mm)											
Mean of diameter (mean ± SD)	9.72 ± 6.73	12.23 ± 5.96	< 0.001	13.01 ± 6.07	11.54 ± 5.55	14.09 ± 6.22	12.59 ± 5.90	12.65 ± 6.10	14.60 ± 6.35	12.83 ± 6.59	0.839
Median of diameter (median ± IQR)	7.22 ± 3.54	9.99 ± 4.08	< 0.001	12.01 ± 4.53	10.10 ± 3.85	13.16 ± 4.49	10.79 ± 4.25	12.01 ± 3.59	14.27 ± 4.75	10.52 ± 9.44	0.624
Size distribution (*N*, %)			< 0.001								0.834
< 4 mm	98 (10.8)	28 (3.8)		1 (0.3)	0 (0.0)	1 (0.6)	1 (0.6)	0 (0.0)	0 (0.0)	1 (1.6)	
≥ 4 mm to < 6 mm	245 (27.0)	118 (16.0)		27 (9.4)	17 (14.0)	10 (6.1)	18 (10.6)	6 (10.3)	3 (5.2)	6 (9.7)	
≥ 6 mm to < 8 mm	181 (20.0)	140 (19.0)		40 (14.0)	22 (18.2)	18 (10.9)	24 (14.1)	9 (15.5)	7 (12.0)	9 (14.5)	
≥ 8 mm to < 15 mm	191 (21.1)	281 (38.1)		123 (43.0)	51 (42.2)	72 (43.6)	72 (42.4)	27 (46.6)	24 (41.4)	26 (41.9)	
≥ 15 mm	192 (21.1)	171 (23.1)		95 (33.3)	31 (25.6)	64 (38.8)	55 (32.3)	16 (27.6)	24 (41.4)	20 (32.3)	

*N/A* not available, *SD* standard deviation, *IQR* interquartile range

^a^ *p*-value reflects the statistical significance of differences of parameters between the multicenter classification dataset (training, validation and testing sets included) and the independent testing set

**Fig. 2:**
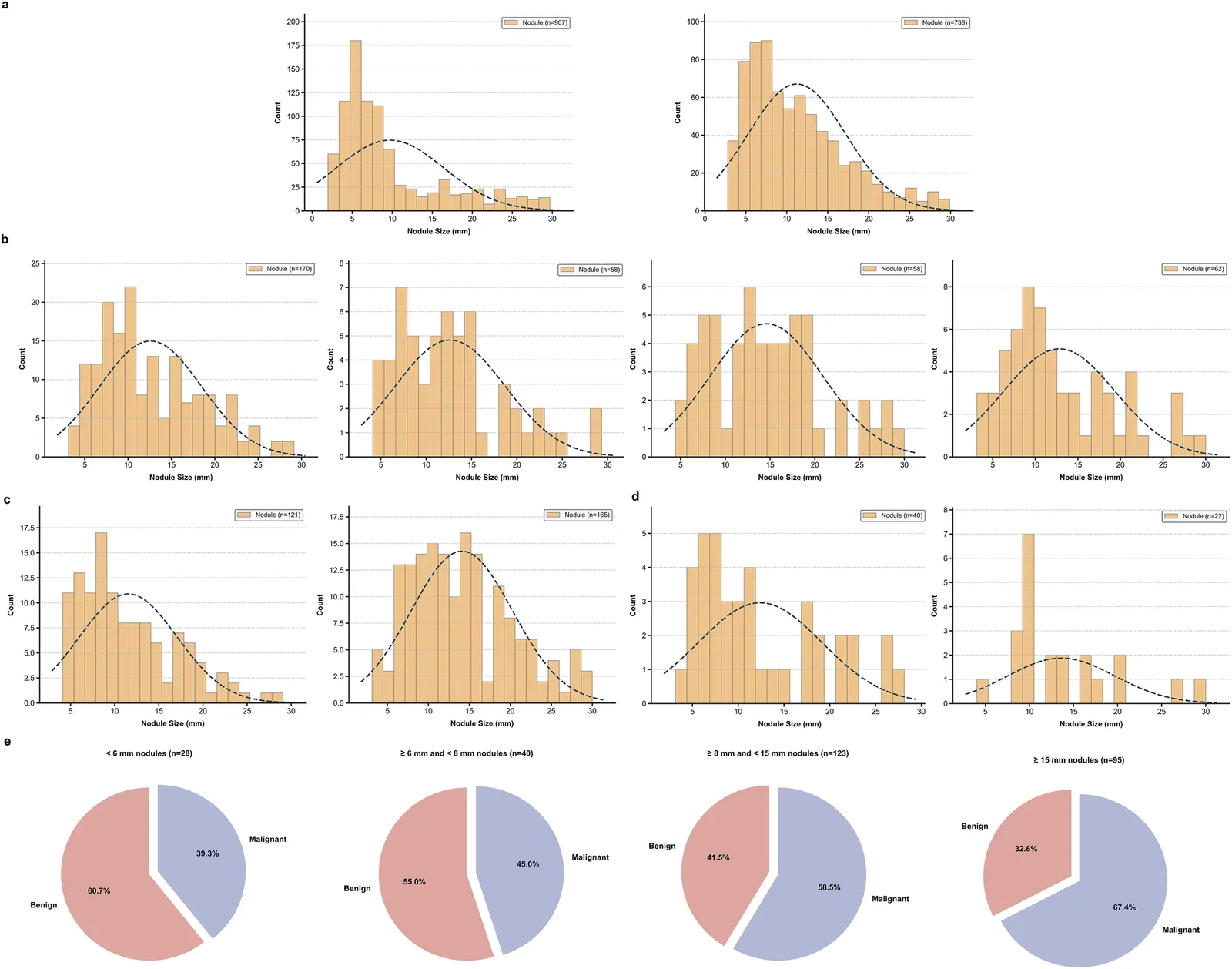
Participants and characteristics of segmental or more proximal airway nodules. **a** Nodule size distributions with the corresponding Gaussian fitted curves in the training (Left) and independent testing datasets (Right) for nodule detection. **b** Nodule size distributions with the corresponding Gaussian fitted curves in the training, validation and testing sets of the multicenter classification dataset and the external independent testing set (from Left to Right). **c** Size distributions of benign (Left) and malignant nodules (Right) with the corresponding Gaussian fitted curves in the multicenter classification dataset. **d** Size distributions of benign (Left) and malignant nodules (Right) with the corresponding Gaussian fitted curves in the external independent testing set. **e** The proportions of malignant nodules with different diameters in the multicenter classification dataset

The training dataset of the detection model contained 907 segmental and more proximal airway nodules (mean diameter 9.72 mm, median 7.22 mm). The independent testing dataset of the detection model consisted of 738 airway nodules (mean diameter 12.23 mm, median 9.99 mm). The multicenter classification dataset included 121 benign (mean diameter 11.54 mm, median 10.10 mm) and 165 malignant (mean diameter 14.09 mm, median 13.16 mm) segmental or more proximal airway nodules for the training, validation and testing of classification model, and the independent testing set of classification model contains 40 benign (mean diameter 12.40 mm, median 10.52 mm) and 22 malignant (mean diameter 13.61 mm, median 11.09 mm) proximal airway nodules (Table 1). Size and location distributions of nodules were displayed in Table 1. Age and nodule size distributions across sets were visualized in Fig. S2 and Fig. 2a, b. The size distribution of benign and malignant nodules of multicenter classification dataset revealed size-dependent proportion variations (Fig. 2c). The size distribution of benign and malignant nodules from the independent testing set of classification model was displayed in Fig. 2d. The proportions of malignant nodules were higher in larger nodules (58.5% for 8-15 mm, 67.4% for ≥ 15 mm) compared to smaller nodules (39.3% for < 6 mm, 45.0% for 6–8 mm) (Fig. 2e).

### Assessment of detection and classification model performance

Our nodule detection algorithm can identify segmental and more proximal nodules, calculate the diameters and output the locations of nodules. It demonstrated good performance on independent testing datasets, achieving a SEN of 73.7%, F1-score of 69.6% and FNR of 26.3% (Table 2). Notably, the SEN value was improved with the increasing size of nodules. For nodules larger than 8 mm, the SEN was 77.2%. These results demonstrated the model’s effectiveness for automated detection and volumetry of airway nodules, with potential to improve diagnostic accuracy in clinical practice.

**Table 2:** The performance of the nodule detection model in the training and independent testing datasets

Variables (%, 95% CI)	Training dataset	Independent testing dataset
Total		
SEN	86.2 (83.8–88.4)	73.7 (70.4–76.9)
F1-score	72.1	69.6
FNR	13.8 (11.6–16.2)	26.3 (23.1–29.6)
< 4 mm		
SEN	75.5 (65.8–83.6)	67.9 (47.6–84.1)
≥ 4 mm to < 6 mm		
SEN	90.6 (86.2–94.0)	67.8 (58.6–76.1)
≥ 6 mm to < 8 mm		
SEN	90.1 (84.7–94.0)	68.6 (60.2–76.1)
≥ 8 mm to < 15 mm		
SEN	89.5 (84.3–93.5)	77.2 (71.9–82.0)
≥ 15 mm		
SEN	79.2 (72.7–84.7)	77.2 (70.2–83.3)

*CI* confidence intervals, *SEN* sensitivity, *FNR* false negative rates

On the negative control set, the model generated a total of only 40 false positive bounding boxes across all 136 negative cases (false positives per scan of 0.29) through applying the exact same pre-defined confidence probability threshold of 0.65 used in the independent testing dataset. Furthermore, case-level analysis revealed that 101 out of the 136 scans (74.3%) contained zero false positive predictions. In comparison with the false positive per scan of 0.77 (281 false positives across 367 scans) on the independent testing dataset, the remarkably low false positives per scan of 0.29 on the negative control set reflects the varying complexity of the image backgrounds. Patients with true proximal airway nodules often present with complex anatomical backgrounds, such as concurrent peri-bronchial inflammation or mucosal irregularities, inherently generating more false positive predictions compared to the anatomically cleaner airways of the negative control set.

Furthermore, repeated scans from the same individuals were filtered, and then the performance of the nodule detection model was re-evaluated on 303 initial baseline scans from 303 patients of the independent testing dataset. The model achieved a lesion-level SEN of 77.6% (95% CI: 74.0–80.8), F1-score of 68.6% and FNR of 22.4% (95% CI: 19.2–26.0), consistent with the performance on the full testing dataset. This finding indicated that including repeated scans from the same individuals didn’t significantly bias the model’s performance.

**Fig. 3:**
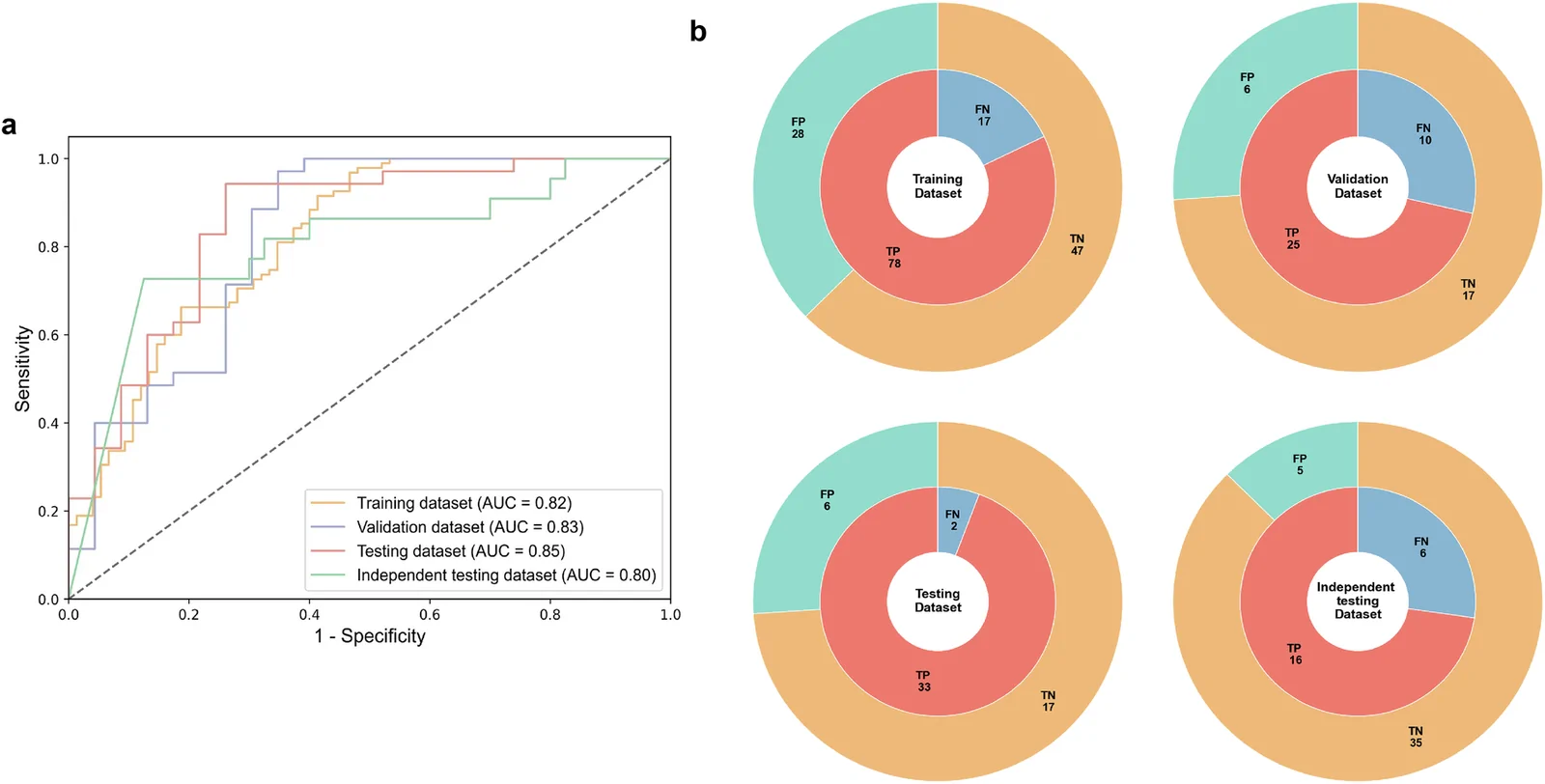
The DL-based nodule classification model for segmental or more proximal airway nodules. **a** The Receiver Operating Characteristic (ROC) curve analysis for the benign-malignant classification model of segmental or more proximal airway nodules. **b** The performance of the benign-malignant classification model in multicenter training, validation, testing and independent testing datasets

The nodule classification model exhibited satisfied SEN (82.1%, 71.4%, 94.3%, 72.7%) and SPC (62.7%, 73.9%, 73.9%, 87.5%) across training, validation, testing and independent testing sets, with AUC values of 0.82 (95% CI 0.74–0.88), 0.83 (95% CI 0.70–0.93), 0.85 (95% CI 0.72–0.94), 0.80 (95% CI 0.68–0.93), respectively (Fig. 3a and Table 3). The model showed a FNR of 27.3% (vs. 17.9% training, 28.6% validation, 5.7% testing) and a FPR of 12.5% (vs. 37.3% training, 26.1% validation, 26.1% testing) in the independent testing set (Fig. 3b and Table 3). These results indicated the model’s superior performance in distinguishing malignant from benign segmental and more proximal airway nodules.

**Table 3:** The performance of the nodule classification model in the multicenter classification dataset and independent testing sets

Variables	Multicenter classification dataset			Independent testing set
(95% CI)	Training set	Validation set	Testing set	
AUC	0.82 (0.74–0.88)	0.83 (0.70–0.93)	0.85 (0.72–0.94)	0.80 (0.68–0.93)
SEN (%)	82.1 (72.9–89.2)	71.4 (53.7–85.4)	94.3 (80.8–99.3)	72.7 (51.8–86.8)
SPC (%)	62.7 (50.7–73.6)	73.9 (51.6–89.8)	73.9 (51.6–89.8)	87.5 (73.9–94.5)
FPR (%)	37.3 (26.1–48.5)	26.1 (9.1–44.0)	26.1 (10.0–46.2)	12.5 (5.5–26.1)
FNR (%)	17.9 (10.6–25.7)	28.6 (14.3–44.5)	5.7 (0.0–14.6)	27.3 (13.2–48.2)
PPV (%)	73.6 (67.2–79.1)	80.7 (67.0–89.5)	84.6 (73.3–91.7)	76.2 (54.9–89.4)
NPV (%)	73.4 (63.5-81.5)	63.0 (48.8–75.2)	89.5 (68.4–97.1)	85.4 (71.6–93.1)
ACC (%)	73.5 (66.2–80.0)	72.4 (59.1–83.3)	86.2 (74.6–93.9)	82.3 (71.0–89.8)

*CI* confidence intervals, *AUC* area under the curve, *SEN* sensitivity, *SPC* specificity, *FPR* false positive rates, *FNR* false negative rates, *PPV* positive prediction value, *NPV* negative prediction value, *ACC* accuracy

### The performance of human-AI interaction

**Fig. 4:**
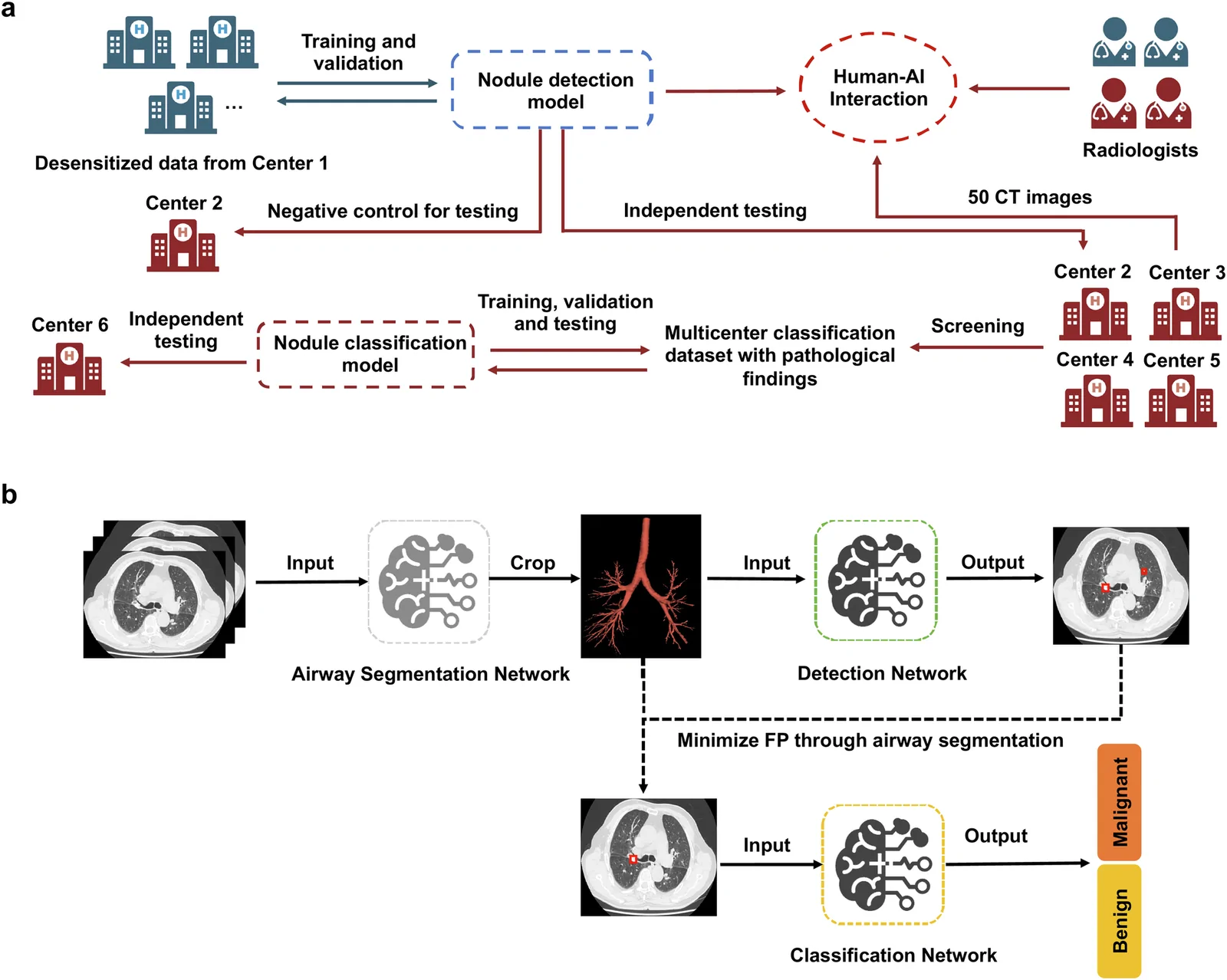
Integrated workflow for the establishment and evaluation of detection and classification models for segmental or more proximal airway nodules. **a** The flowchart for training, validation and testing of detection and classification models and human-AI interaction of the nodule detection model. **b** Integrated workflow for the establishment of nodule detection and classification networks

Integrated workflow of the establishment and evaluation of detection and classification models for segmental and more proximal airway nodules was visualized in Fig. 4. Human-AI interaction was employed to evaluate the actual utility of the nodule detection model in assisting image interpretation for radiologists. The performance and average time consumption of junior and senior radiologists with or without AI assistance were displayed in Table 4. Notably, compared with radiologists without AI assistance, the probability of nodule detection and F1-score were significantly increased from 39.5% and 49.4% to 72.9% and 77.2%, respectively (*p* = 0.0341, 0.0252), while missed diagnosis rates were remarkably reduced for junior and senior radiologists with AI assistance from 60.5% to 27.1% (*p* = 0.0341). Furthermore, mean times of nodule detection for radiologists were significantly shortened with AI assistance from 83.8 s to 61.8 s (*p* = 0.0471). These findings indicated that the proposed AI model could enhance diagnostic capabilities and reduce the diagnosis time of radiologists in a clinical scenario.

**Table 4:** The performance and average time consumption of radiologists in nodule detection with or without AI assistance

Variables	SEN (95% CI)	F1-score	FNR (95% CI)	Mean times (s)
Junior 1-NO AI	25.2 (18.3–33.1)	37.7	74.8 (66.9–81.7)	104.6
Junior 2-NO AI	31.5 (24.0–39.8)	43.1	68.5 (62.0–76.0)	86.2
Senior 1-NO AI	57.3 (48.8–65.6)	63.6	42.7 (34.4–51.2)	74.4
Senior 2-NO AI	44.1 (35.8–52.6)	53.2	55.9 (47.4–64.2)	69.9
Mean without AI	39.5	49.4	60.5	83.8
Junior 1-AI	72.7 (64.7–79.8)	78.2	27.3 (20.2–35.3)	64.7
Junior 2-AI	72.0 (63.9–79.2)	76.9	28.0 (20.8–36.1)	78.9
Senior 1-AI	64.3 (55.9–72.2)	73.0	35.7 (27.8–44.1)	53.2
Senior 2-AI	82.5 (75.3–88.4)	80.8	17.5 (11.6–24.7)	50.5
Mean with AI	72.9	77.2	27.1	61.8
*p*-value	0.0341	0.0252	0.0341	0.0471

*AI* artificial intelligence, *SEN* sensitivity, *CIs* confidence intervals, *FNR* false negative rates

## Discussion

Our study proposed a DL-based CAD system for early detection and risk stratification of segmental or more proximal airway nodules on chest LDCT images, validated on multicenter retrospective datasets. This automated model swiftly detects and accurately classifies segmental or more proximal airway nodules as a second-reading tool, thereby reducing missed/delayed diagnoses, improving early detection rates of malignant nodules, and preventing unnecessary invasive checkups while enabling early medical intervention.

**Fig. 5:**
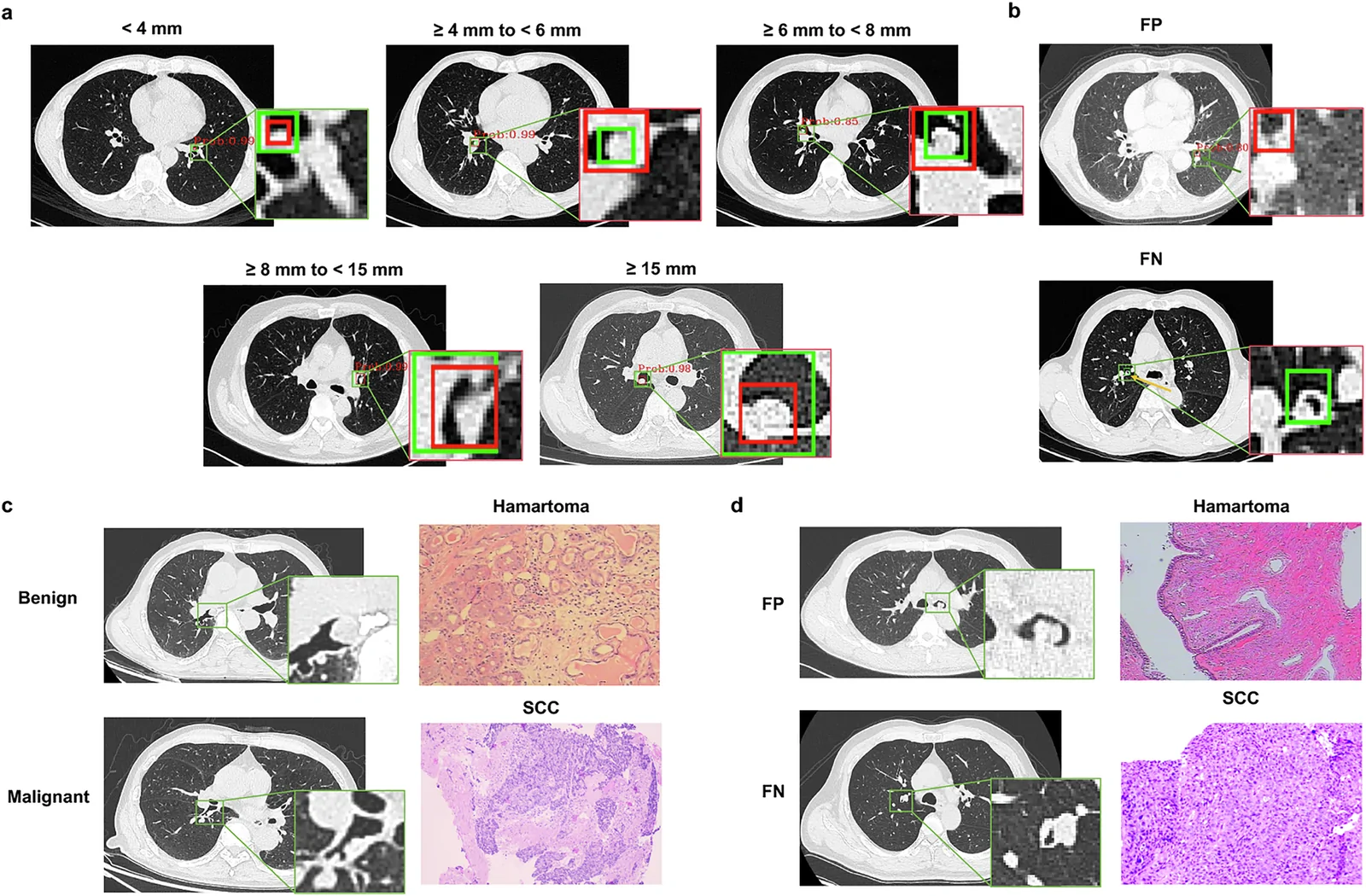
The application examples of the proposed nodule detection and classification models. **a** The examples of nodule detection models on segmental or more proximal airway nodules with different diameters (The green box represents the gold standard box, and the red box represents the model prediction box). The sizes of detected nodules were 3.86, 5.89, 6.24, 13.31 and 24.57 mm from left to right and from top to bottom. The corresponding locations were the anterior inner basal segment of the lower lobe of the left lung, the dorsal segment of the lower lobe of the right lung, the outer segment of the middle lobe of the right lung, the posterior apical segment of the upper lobe of the left lung, and the middle segment bronchus of the right lung, respectively. **b** The cases of false positives (FP) originated from tracheal septa and false negatives (FNs) due to small airway nodules for the nodule detection model. **c** The examples of the nodule classification model on benign and malignant segmental or more proximal airway nodules. **d** The cases of FP and FN for the nodule classification model. (Upper) A 50-year-old male patient pathologically confirmed with hamartoma was misclassified as malignant, attributed to irregularly shaped and narrow-based airway lesions with exophytic cauliflower-like growth patterns. (Lower) A 59-year-old male patient pathologically confirmed with squamous cell carcinoma (SCC) was misclassified as benign due to small airway lesions

**Fig. 6:**
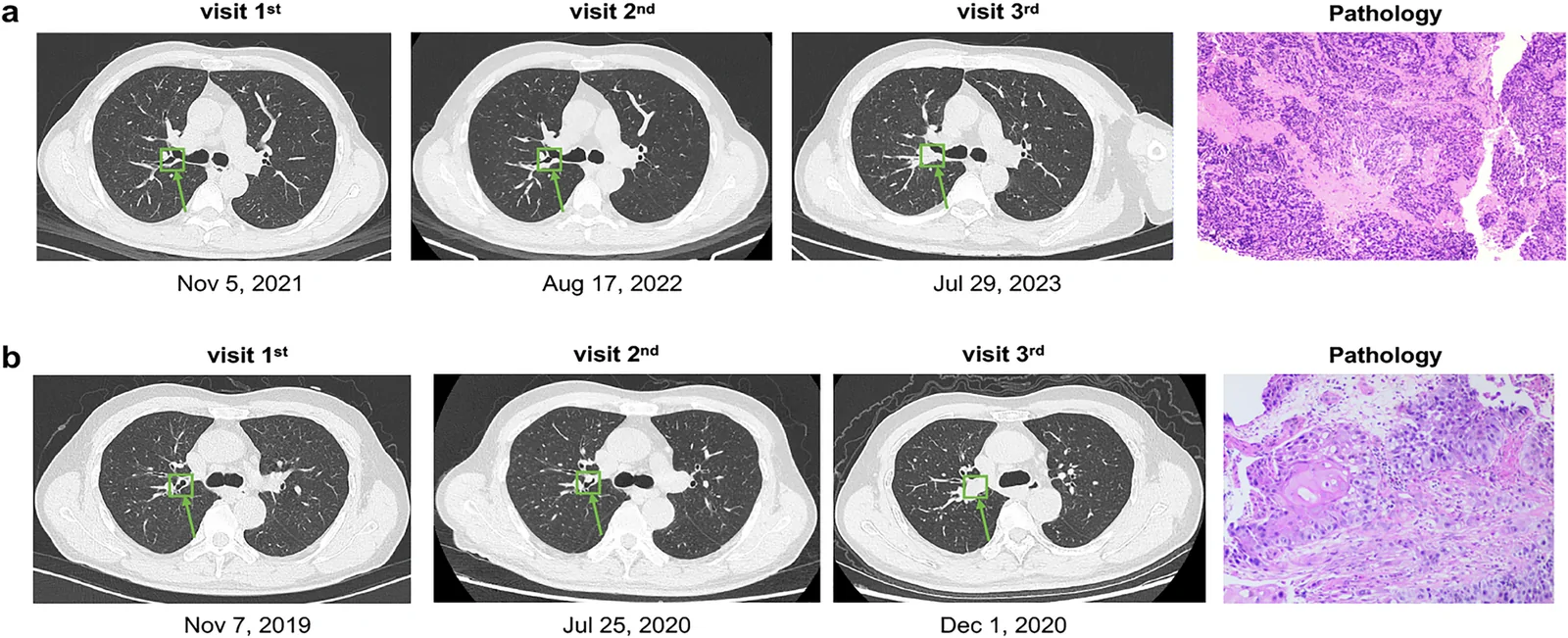
Representative cases missed by radiologists without artificial intelligence (AI) assistance, but detected earlier by our AI model. **a** Representative CT slices of missed segmental or more proximal airway nodules (Left), and the corresponding pathological findings from a 55-year-old male patient with lung cancer (Right). The malignant nodule was identified by radiologists during the third follow-up on July 29, 2023, but was detected by our AI model during the initial scan on November 5, 2021. **b** Representative CT slices of missed segmental or more proximal airway nodules (Left), and the corresponding pathological findings from a 67-year-old male patient with lung cancer (Right). The malignant nodule was identified by radiologists during the third follow-up on December 1, 2020, but was detected by our AI model during the second scan on July 25, 2020

Leveraging airway segmentation and transfer learning, the proposed detection model identified segmental and more proximal airway nodules at an early stage with 73.7% SEN in external validation, and output the diameter and location of nodules with automated bounding boxes. Detection examples across nodule with different diameters were displayed in Fig. 5a. Additionally, two representative cases that were missed by radiologists until the third follow-up, potentially attributed from subjective neglect of airway lesions or interpreter fatigue, were detected earlier by our AI model (Fig. 6). Notably, human-AI interaction demonstrated that junior and senior radiologists with the assistance of nodule detection model showed enhanced detection accuracy with shortened diagnosis time for segmental or more proximal airway nodules on chest CT scans, suggesting its promising clinical utility as a second-reading tool.

Inevitably, conditions of false positives (FPs) and false negatives (FNs) emerged, as illustrated in Fig. 5b. The majority of FP originated from tracheal septa and irregular hardening/calcification of the airway wall. Peribronchial small vessels were also occasionally misclassified as airway nodules. In clinical practice, these FPs could be minimized by tracing the airway anatomy across consecutive slices. Developing detection models incorporating anatomical priors to differentiate airway nodules from tracheal septa would reduce FPR and enhance clinical utility. FNs primarily involved small airway nodules with low contrast to the background that challenged both AI models and radiologists.

With regards to the classification and management of airway nodules, Lung-RADS v1.0 and v1.1 in 2019 firstly advocated the standardized criteria, later refined in Lung-RADS v2022 [17, 29]. Lung-RADS v2022 stratifies malignant risk based on initial CT characteristics (location, morphology and number of nodules) and persistence in the follow-up CT, recommending either long/short-term follow-up or clinical diagnostic evaluation. Persistent suspicious airway nodules at 1 or 3-month follow-up CT are considered highly suspicious and warrant bronchoscopy. Note that it is challenging to differentiate benign from malignant segmental or more proximal airway nodules without benign features on initial LDCT, with follow-up intervals differing between Lung-RADS v2022 and the National Comprehensive Cancer Network management guidelines [30].

We developed a DL-based classification model to characterize segmental and more proximal airway nodules from a single LDCT scan (Fig. 5c), assisting radiologists in management decisions and enabling prompt clinical intervention for high-risk airway nodules to facilitate early diagnosis and treatment of lung cancer. However, cases of FP and FN remain (Fig. 5d). FP cases typically involved irregularly shaped, narrow-based benign lesions demonstrating exophytic cauliflower-like growth patterns. The majority of FNs occurred with small lesions, cylindrically-shaped lesions mimicking mucus plugs, and well-circumscribed malignant lesions with regular contours.

A prior single-center retrospective study has reported an effective AI system aiding airway nodule detection in LCS protocols, based on CTs from 160 patients with airway nodules (82 tracheobronchial tumors, 78 non-tumorous nodules) and 160 matched negative controls without airway nodules [21]. Our work developed both nodule detection and benign-malignant classification models using relative larger-sample data from multiple centers, including 1107 chest LDCT scans and 1813 airway nodules, of which 187 nodules were pathologically confirmed as malignancy. Notably, unlike the published study, our work focuses on endobronchial lesions without lung parenchyma involvement and excludes indirect imaging signs of lung cancer, such as obstructive central lesions, since radiologists seldom miss these lesions. Furthermore, human-AI interaction demonstrated good actual utility of the nodule detection model, which draws the radiologist’s attention to visually elusive proximal airway nodules that might otherwise be overlooked due to interpreter fatigue. Additionally, a nodule classification model was developed to provide a second opinion of diagnosis for radiologists to shorten follow-up time and enable early medical intervention.

Some limitations remain. First, the limited sample size of annotated CT scans might constrain the generalizability of the classification model. Although the nodule classification model was trained, validated and tested using multicenter data and evaluated in an external independent testing set, the sample size of the multicenter classification dataset was relatively small, and the external cohort was derived from a single center. Therefore, a larger number of examples for model training and validation is warranted, as well as a larger sample size and multicenter external validation. Second, class imbalance and unclear representativeness of the multicenter classification dataset might influence the clinical utility of the classification model. Prospective cohort studies are needed to validate the clinical utility of the system in the real world. Third, FN cases of the classification model were unavoidable. The incorporation of clinical data, molecular biomarkers and follow-up information could enhance model accuracy. Fourth, the proposed model was adapted for segmental and more proximal airway nodules, while its capability to detect and classify subsegmental or more distal small airway nodules requires enhancement.

Our automated detection model assists radiologists in swiftly and accurately identifying segmental and more proximal airway nodules on chest LDCT, reducing missed diagnoses. The DL-based risk stratification model improves early recognition of malignant airway nodules on initial screening CTs, preventing diagnostic delays and improving clinical outcomes of lung cancer originating from airway lesions. This system is expected to significantly advance the early detection and diagnosis of segmental and more proximal airway nodules, thereby guiding early medical treatment.

## Supplementary information

ELECTRONIC SUPPLEMENTARY MATERIAL

## Data Availability

All the original data supporting this paper are available on request to the corresponding authors.

## Abbreviations

AI: Artificial intelligence
CI: Confidence intervals
CT: Computed tomography
DL: Deep learning
FNR: False negative rates
FPR: False positive rates
LCS: Lung cancer screening
LDCT: Low-dose computed tomography
Lung-RADS: The Lung Imaging Reporting and Data System
NPV: Negative prediction value
PPV: Positive prediction value
SCC: Squamous cell carcinoma
SEN: Sensitivity
SPC: Specificity
