# Supplementary material for: Deep learning-based detection and classification of airway nodules on CT for early medical intervention
Source: Insights Imaging. 2026 Nov 5;17:282. doi: 10.1186/s13244-026-02446-7 (PMC13740212; doi:10.1186/s13244-026-02446-7)
Supplement: Supplementary file 1 [file 13244_2026_2446_MOESM1_ESM.docx]

Supplemental materials for

Deep Learning-Based Detection and Classification of Airway Nodules on CT for Early Medical Intervention

Materials and Methods

*Establishment of Detection Model*

Pulmonary airway segmentation

For pulmonary airway segmentation, the WingsNet [22] was adopted with a novel training strategy. The WingsNet was constructed upon an UNet framework, renowned for its ability to capture fine anatomical details and produce precise segmentations. The network architecture is composed of multiple ConvBlocks, which are the fundamental building units of the network. Each ConvBlock consists of a convolutional layer followed by normalization and activation layers. Furthermore, the model incorporated group supervision, which divides ConvBlocks into distinct groups that are trained with individual loss functions, enhancing the learning process for shallow layer features.

In the training, the WingsNet utilized a General Union loss to handle intra-class imbalances, ensuring that both large and small airways were accurately segmented. Moreover, we introduced two key improvements. First, we implemented dynamical voxel reweighting based on the local imbalance and the distance from the central line (the closer to the central line, the higher the weight). Second, small airway was oversampling combined with a skeleton-based hard case mining strategy.

Nodule detection algorithm

For nodule detection, we employed the SCPM-Net (detailed in Figure S1a) [23] enhanced with coordinate attention [24] and multiscale attention [25] modules.

The SCPM-Net utilized an encoder-decoder architecture to generate a center point map (MC), a radius map (MR), and an offset map (MO). This 3D bounding sphere representation (centroid, radius, local offset) better aligns with clinical annotations and provides a more natural representation of nodules. The network incorporates a positive center-points selection and matching process that enhances robustness by focusing on the key points around the nodule centroid. Furthermore, SCPM-Net eliminates predefined anchor boxes and manual parameter tuning, which are typically required in anchor-based detection methods, thereby reducing complexity and computational load.

Coordinate attention is a novel attention mechanism proposed for mobile networks that enhances model performance by integrating positional information into channel attention. Unlike traditional channel attention which uses two-dimensional (2D) global pooling, coordinate attention employs two parallel 1D feature encoding processes to aggregate features along spatial dimensions, capturing long-range dependencies while preserving precise positional information. This generates direction- and position-sensitive attention maps.

The convolutional quadruplet attention module (CQAM) as a key component of the multiscale attention block, was designed to capture cross-dimensional interactions within feature maps, enhancing the model’s ability to focus on informative features for accurate nodule identification. The CQAM computes attention weights through four specialized branches that focus on different aspects of the data. Requiring minimal additional learnable parameters, CQAM enhances detection sensitivity without substantially increasing the network complexity.

Training of nodule detection method

In the training, the SCPM-Net adopted a novel center-points matching strategy, which dynamically assigns positive, negative, and ignored labels to improve the network’s ability to identify nodule centroids. Besides, the sphere-based intersection over union (SIoU), online hard example mining (OHEM) and re-focal loss were calculated as the loss functions. The SIoU incorporates geometric measures including sphere overlap, center point distance, and intersection angles to provide geometrically meaningful training signals. The OHEM and re-focal los address class imbalance and focus the training on harder examples, improving point assignment accuracy and detection performance.

Furthermore, due to limited airway nodule images, we initially trained the model on lung-field nodules, and then applied JAN [26] for domain adaptation to airway images. The JAN is a novel deep transfer learning approach for unsupervised domain adaptation. JAN addressed the challenge of domain shift by aligning the joint distributions of activations from multiple layers of a deep neural network, which are domain-specific. This alignment was achieved via Joint Maximum Mean Discrepancy (JMMD), which operates on the kernel mean embeddings of empirical joint distributions.

JAN extends upon previous deep adaptation networks by incorporating JMMD to measure and minimize the discrepancy between the joint distributions of the source and target domains. The framework employs an adversarial training strategy to maximize JMMD, which in turn makes the source and target domain distributions more distinguishable. This adversarial process is particularly effective in creating robust features that are invariant to the differences between domains. Note that a key contribution of JAN is its scalable learning ability by providing a linear-time unbiased estimate of JMMD. This allows for efficient computation of gradients using back-propagation, which is crucial for training deep networks.

Notably, our detection model defined the target lesion by the 3D bounding box that occupies only a minute fraction of the entire 3D volume. The vast majority of the remaining 3D space was regarded as negative samples. This background space includes normal airway walls, tracheal septa, peri-bronchial vessels, and healthy tissue. During the training process, the model dynamically performs negative sampling across this extensive background volume to compute the classification loss. This allows the network to effectively learn the 3D spatial context and differentiate true airway nodules from normal anatomical structures.

*Establishment of Classification Model*

The input 3D CT images were initially cropped with a 15-pixel expansion around detection box, to capture of additional contextual information related to the tracheal nodules. Subsequently, the images were resampled and resized to a standardized size of 224×224×224 voxels for network input. To enhance the model’s resolution and processing capability, the 3D SEResNeXt101 network was employed (Figure S1b) [27; 28]. This network incorporated an advanced 3D CNN-the SE module. The SE module adaptively learned to adjust channel weights, enabling the network to focus on critical features, thus enhancing its representational power. Built upon the ResNeXt architecture, SEResNeXt101 enhances capabilities of cross-scale feature extraction and 3D medical image analysis through optimized network parameters. During training, a batch size of 12 was set, with an initial learning rate of 1e-3, and a CosineAnnealingLR was applied to adjust the learning rate, with a minimum value of 1e-6. To further improve the model's robustness and generalization capacity, a series of data augmentation techniques were implemented, including random 90-degree rotations, random flips, and the addition of Gaussian noise.

Figure S1. The network architectures for nodule detection and classification models.

a. The network architecture of the sphere-based center-points matching network (SCPM-Net) for nodule detection.

b. Schematic diagram of nodule classification model architecture.

Figure S2. Age distribution of participants in datasets for nodule detection and classification.

a. Age distribution with the corresponding Gaussian fitted curves of participants in the training (Left) and independent testing datasets (Right) for nodule detection.

b. Age distribution with the corresponding Gaussian fitted curves of participants in the training (Top left), validation (Top right) and testing sets (Bottom left) of multicenter classification dataset, and the independent testing set (Bottom right).

Table S1. Construction and labeling of datasets.

| Datasets | Dataset 1 | Dataset 2 | Dataset 3 | | Dataset 4 | Dataset 5 | Dataset 6 | |
| --- | --- | --- | --- | --- | --- | --- | --- | --- |
| Sources | Center 1 | Center 2 and 3 | Center 2, 3, 4 and 5 | | Center 2 | Center 2 | Center 6 | |
| Number of total patients | 926 | 1487 | 28033 | | 106 | 136 | 62 | |
| Number of enrolled participants | 436 | 180 | 123 | | 106 | 136 | 62 | |
| Number of enrolled chest CT scans | 436 | 187 | 180 | | 106 | 136 | 62 | |
| Number of enrolled airway nodules | 907 | 357 | 381 | 180 | 106 | 0 | 25 | 37 |
| Characteristics | Multicenter desensitized data | Proximal airway nodules from manual retrieval in PACS | Total proximal airway nodules from pathologically confirmed patients with benign/malignant lung neoplasms/lesions | Pathologically confirmed benign/malignant proximal airway nodules | Mucous plugging | Negative airway nodules | Pathologically confirmed benign/malignant proximal airway nodules | Mucous plugging |
| Roles | Training dataset for nodule detection model | Independent testing dataset for nodule detection model | |  | Negative controls | Negative control set for testing of nodule detection model | Independent testing dataset for nodule classification model | |
|  |  |  |  | Training, validation and testing sets of nodule classification model | |  |  |  |

Table S2. CT equipment and parameters of scanning and reconstruction across five hospitals in Hunan Province.

| Hospital | CT equipment | Tube voltage/kVp | Tube current/mAs | Pitch/mm | Scan slice thickness/mm | Reconstructed slice thickness/mm | Matrix | Field of view (mm × mm) | Slice increment/mm |
| --- | --- | --- | --- | --- | --- | --- | --- | --- | --- |
| Center 2 | Philips Spectral CT | 120 | 137 | 0.600 | 1.0 | 1.0 | 1024×1024 | 350×350 | 0.5 |
| Center 3 | Philips Brilliance ICT | 120 | 150 | 0.586 | 0.625 | 1.0 | 1024×1024 | 350×350 | 0.5 |
| Center 4 | TOSHIBA Aquilion PRIME 64 | 120 | 350 | 0.641 | 0.5 | 2.0 | 512×512 | 400×400 | 0.5 |
| Center 5 | General Electric VCT | 120 | 450 | 1.375 | 1.25 | 5.0 | 512×512 | 500×500 | 1.25 |
| Center 6 | GE Revolution CT | 120 | 400 | 0.992 | 0.625 | 0.625 | 1024×1024 | 320×320 | 1.0 |

Table S3. Time consumption of radiologists in nodule detection with or without AI assistance.

| Patients | Junior 1-NO AI (s) | Junior 2-NO AI (s) | Senior 1-NO AI (s) | Senior 2-NO AI (s) | Junior 1-AI (s) | Junior 2-AI (s) | Senior 1-AI (s) | Senior 2-AI (s) |
| --- | --- | --- | --- | --- | --- | --- | --- | --- |
| 1 | 56 | 191 | 125 | 51 | 165 | 89 | 91 | 69 |
| 2 | 178 | 160 | 74 | 158 | 160 | 77 | 51 | 66 |
| 3 | 53 | 63 | 73 | 52 | 133 | 88 | 38 | 39 |
| 4 | 63 | 68 | 31 | 43 | 116 | 45 | 54 | 45 |
| 5 | 100 | 89 | 65 | 69 | 116 | 69 | 35 | 56 |
| 6 | 199 | 119 | 90 | 62 | 115 | 84 | 55 | 26 |
| 7 | 161 | 189 | 76 | 74 | 114 | 81 | 59 | 66 |
| 8 | 39 | 118 | 86 | 62 | 105 | 77 | 51 | 54 |
| 9 | 187 | 163 | 45 | 71 | 92 | 90 | 33 | 26 |
| 10 | 82 | 87 | 43 | 157 | 88 | 118 | 90 | 93 |
| 11 | 230 | 126 | 89 | 79 | 80 | 105 | 142 | 94 |
| 12 | 59 | 76 | 60 | 50 | 79 | 94 | 50 | 38 |
| 13 | 340 | 65 | 54 | 70 | 70 | 66 | 81 | 99 |
| 14 | 171 | 148 | 107 | 67 | 65 | 111 | 158 | 132 |
| 15 | 183 | 151 | 69 | 45 | 62 | 87 | 65 | 89 |
| 16 | 130 | 73 | 92 | 47 | 61 | 89 | 67 | 36 |
| 17 | 90 | 88 | 94 | 49 | 60 | 69 | 56 | 78 |
| 18 | 146 | 69 | 80 | 53 | 59 | 71 | 41 | 25 |
| 19 | 51 | 51 | 80 | 55 | 59 | 104 | 59 | 79 |
| 20 | 225 | 96 | 87 | 87 | 58 | 63 | 45 | 45 |
| 21 | 162 | 122 | 79 | 98 | 57 | 74 | 27 | 36 |
| 22 | 89 | 75 | 71 | 77 | 54 | 58 | 62 | 48 |
| 23 | 34 | 50 | 110 | 86 | 54 | 69 | 71 | 71 |
| 24 | 127 | 83 | 65 | 51 | 52 | 75 | 63 | 28 |
| 25 | 52 | 86 | 84 | 58 | 50 | 116 | 40 | 31 |
| 26 | 52 | 33 | 117 | 62 | 50 | 75 | 99 | 74 |
| 27 | 127 | 41 | 67 | 82 | 49 | 85 | 40 | 16 |
| 28 | 54 | 61 | 57 | 99 | 48 | 54 | 32 | 39 |
| 29 | 107 | 117 | 65 | 80 | 48 | 57 | 18 | 22 |
| 30 | 110 | 56 | 80 | 54 | 48 | 107 | 48 | 48 |
| 31 | 81 | 45 | 70 | 43 | 47 | 64 | 42 | 35 |
| 32 | 43 | 87 | 89 | 79 | 47 | 85 | 38 | 54 |
| 33 | 55 | 58 | 61 | 54 | 43 | 104 | 59 | 70 |
| 34 | 75 | 71 | 35 | 55 | 42 | 94 | 35 | 44 |
| 35 | 102 | 119 | 63 | 85 | 40 | 89 | 34 | 28 |
| 36 | 103 | 104 | 51 | 48 | 39 | 66 | 69 | 68 |
| 37 | 115 | 110 | 93 | 65 | 38 | 120 | 50 | 76 |
| 38 | 203 | 138 | 108 | 43 | 57 | 57 | 23 | 15 |
| 39 | 44 | 44 | 83 | 74 | 33 | 65 | 57 | 36 |
| 40 | 57 | 48 | 46 | 50 | 33 | 60 | 39 | 43 |
| 41 | 246 | 96 | 96 | 87 | 32 | 63 | 27 | 28 |
| 42 | 63 | 69 | 60 | 97 | 31 | 47 | 36 | 29 |
| 43 | 45 | 65 | 74 | 86 | 31 | 70 | 26 | 44 |
| 44 | 46 | 38 | 45 | 70 | 50 | 68 | 30 | 25 |
| 45 | 51 | 40 | 31 | 93 | 90 | 56 | 52 | 48 |
| 46 | 63 | 57 | 100 | 86 | 40 | 110 | 52 | 21 |
| 47 | 22 | 48 | 54 | 72 | 27 | 54 | 30 | 24 |
| 48 | 31 | 63 | 55 | 40 | 24 | 86 | 33 | 68 |
| 49 | 63 | 48 | 119 | 80 | 42 | 88 | 73 | 61 |
| 50 | 62 | 48 | 74 | 42 | 82 | 50 | 32 | 39 |
